# A Pilot Clinical Trial of a Group Coping Intervention for English and Spanish Speakers with Chronic Graft-Versus-Host Disease

**DOI:** 10.21203/rs.3.rs-8564526/v1

**Published:** 2026-02-09

**Authors:** Joely A. Centracchio, Johan Euceda, Sasha Boyers, Anna Barata, Jacqueline Montano, Christopher Michelini, Hanna Culang, Elisabeth Henley, Andrea Pineda, Adriana Alvarez, Meilin Diaz-Paez, Trent P. Wang, Ashley M. Nelson, Areej El-Jawahri, Lara Traeger

**Affiliations:** University of Miami; University of Miami; University of Miami; Massachusetts General Hospital; Sylvester Comprehensive Cancer Center; University of Miami; University of Miami; University of Miami; University of Miami; Sylvester Comprehensive Cancer Center; Sylvester Comprehensive Cancer Center; Sylvester Comprehensive Cancer Center; Massachusetts General Hospital; Massachusetts General Hospital; University of Miami

**Keywords:** Graft vs Host Disease, Coping Skills, Health-Related Quality Of Life, Allografts

## Abstract

**Background:**

Chronic graft-versus-host disease (cGVHD) affects up to 40% of allogeneic hematopoietic cell transplant (HCT) survivors, producing lasting physical and psychosocial sequelae. Despite their high symptom burden, survivors with cGVHD are rarely included in survivorship intervention research. This pilot study evaluated the feasibility and acceptability of a group-based coping intervention for patients with cGVHD (Horizons program), delivered in English and Spanish to patients in South Florida, USA to inform larger-scale testing.

**Methods:**

We conducted a single-arm trial of the Horizons program, enrolling adults with moderate or severe cGVHD from an academic medical center. Enrollment and group assignment were stratified by language. Participants engaged in eight weekly 90-minute videoconference sessions co-led by a transplant clinician and behavioral health expert (5–6 participants per group). Feasibility benchmarks included ≥50% enrollment, ≥80% attendance, and ≥80% retention. Exit interviews were analyzed using content analysis to assess acceptability.

**Results:**

From December 2023 to August 2024, 21 of 40 approached patients enrolled (52.5%). The sample (median age 60) was 71% male and 71% Hispanic/Latino; 11 participated in English and 10 in Spanish. Nineteen participants (90.5%) attended ≥4 sessions, and 95.2% completed follow-up. Treatment fidelity averaged 96.7%. Qualitative feedback underscored increased support, self-efficacy, and connection. Participants emphasized peer validation, the development of coping skills, and comfort in discussing their experiences.

**Conclusion:**

Horizons demonstrated high feasibility, excellent fidelity, and acceptability among diverse, English- and Spanish-speaking cGVHD survivors, supporting further evaluation in larger, multisite trials.

## INTRODUCTION

Allogeneic hematopoietic stem cell transplantation (HCT) is an intensive, but potentially curative treatment for hematologic malignancies (e.g., acute myeloid leukemia [AML], acute lymphoblastic leukemia [ALL], myelodysplastic syndromes [MDS]). Since 2017, more than 42,000 hematopoietic allogeneic cell transplants have been carried out worldwide each year, offering benefits such as improved survival and durable disease control [1]. Despite these impacts on survivorship, patients remain at significant risk of late effects and long-term complications, most notably chronic graft-versus-host disease (cGVHD), detrimentally affecting morbidity, quality of life (QOL), and survivorship [2–6]. cGVHD is a significant inflammatory complication that targets multiple organ systems, most commonly involving the skin, oral cavity, and eyes [7–8]. cGVHD develops in up to 40% of HCT survivors, typically manifesting between three and 18 months after transplantation [9]. The clinical course is highly variable, ranging from mild to severe, and is often accompanied by substantial symptom burden that impairs physical functioning, energy levels, and activities of daily living [10]. Patients with moderate to severe cGVHD are particularly vulnerable to pain, fatigue, sleep disturbances, and cognitive difficulties, further exacerbating QOL impairments [11].

Corticosteroids remain the gold standard for cGVHD treatment but frequently provide only partial symptom relief and may introduce additional adverse effects, complicating disease management [5, 12–13]. As a result, patients often require complex daily regimens of systemic and topical immunosuppressive or anti-inflammatory medications, making long-term adherence a persistent challenge [12, 14]. In parallel with physical symptoms, psychological distress is common: approximately half of individuals with cGVHD report depressive symptoms, and nearly one-third experience anxiety [3, 6, 11].

Effective self-management, including medication adherence, symptom monitoring, self-care, and coping strategies, is essential for optimizing post-HCT outcomes and overall well-being [15]. However, access to survivorship care is often limited by the small number of transplant centers and geographic barriers, particularly for rural patients, leaving many immunocompromised survivors vulnerable to ongoing complications [4, 14, 16]. Together, these challenges underscore the critical need for supportive interventions tailored to help long-term HCT survivors manage the complex and disruptive impact of cGVHD [6, 17].

To address this need, we previously carried out a single-center pilot randomized clinical trial to assess the feasibility and preliminary efficacy of a multidisciplinary group coping skills program (Horizons) compared to minimally enhanced usual care in 80 adults with moderate or severe cGVHD [18]. Individuals assigned to the Horizons group participated in eight weekly group sessions, which were co-facilitated by a transplant clinician and a behavioral health specialist via videoconference. The Horizons program combined education about cGVHD symptoms and treatment options with the teaching and practice of coping strategies for managing daily challenges related to cGVHD in a supportive group environment. Findings showed the feasibility of recruitment, session attendance, and retention, along with the Horizons program’s acceptability and promise for enhancing quality of life and reducing symptoms of depression and anxiety [18,19]

Yet, this prior work was limited by a single-site, English language-only sample with restricted racial and ethnic diversity. The objective of the present mixed-methods pilot study was to expand our understanding of intervention feasibility and acceptability in a more diverse cGVHD patient sample, to support a future larger-scale efficacy trial, and to inform any refinements to the study protocol prior to trial conduct. We hypothesized that English- and Spanish-language delivery of the Horizons program would be feasible, based on enrollment, attendance, intervention fidelity, and retention rates, and acceptable (primary outcomes). The findings of this work will inform future research to tailor and deliver supportive care interventions for diverse post-transplant survivors managing the challenges of cGVHD.

## METHODS

### Design

We conducted a single-arm pilot clinical trial of the Horizons program for patients with moderate or severe cGVHD (n=21) (ClinicalTrials.gov identifier: NCT06160986). The study was conducted at the University of Miami/Sylvester Comprehensive Cancer Network in Miami-Dade County, Florida, USA, where the majority of residents identify as Hispanic/Latino. The Institutional Review Board approved the study protocol; all patients provided informed consent.

#### Participants

Eligible patients were adults aged ≥21 years who underwent allogeneic HCT, experiencing moderate or severe cGVHD as graded by their transplant oncologist using NIH consensus criteria, currently receiving care at the Sylvester Comprehensive Cancer Center Adult Stem Cell Transplantation Program, and able to participate in an English- or Spanish-language intervention [20]. Patients meeting the inclusion criteria were eligible regardless of the time since cGVHD diagnosis. The age criterion allowed for focused recruitment among adults who are responsible for their own medical care. Patients were excluded if they had a comorbid condition or cognitive impairment that the treating clinician believed would prohibit informed consent or participation.

### Procedures

Study staff reviewed the transplant clinic outpatient schedule and electronic health records to identify potentially eligible patients. They then contacted the transplant oncologist to confirm moderate or severe cGVHD status and request permission to approach the patients about participation. Bilingual study staff recruited consecutively eligible patients in English and Spanish until we reached our approximate target sample (i.e., 10 English-speaking and 11 Spanish-speaking). Groups were stratified by language (i.e., 2 English-led and 2 Spanish-led groups). The sample size enabled us to assess feasibility, acceptability, and pre- to postintervention change in outcomes across the total sample. We obtained written or electronic informed consent from all participants before administering the baseline self-report assessment. All patient-facing materials utilized for consent and recruitment were professionally translated. After completion of the baseline survey, each participant was invited to participate in the Horizons program, stratified by preferred language (English or Spanish).

#### The Horizons program Intervention.

The Horizons program draws upon QOL research in HCT recipients [2–3, 6], an established framework for chronic illness self-management [15], and evidence-based psychosocial techniques. These techniques included elements from mindfulness practices (e.g., acceptance), cognitive-behavioral approaches (e.g., behavioral activation), and positive psychology (e.g., meaning-making) [18]. Additionally, input from patient and caregiver stakeholder groups informed the program’s design [17].

The program includes eight weekly sessions, each lasting 90 minutes, conducted securely via videoconference. Groups consisted of four to six participants and were co-facilitated by a transplant clinician (i.e., transplant oncologist or nurse practitioner) alongside a behavioral health professional (i.e., social worker or psychologist). All participants received a workbook detailing all session content and access to recorded mindfulness exercises. The workbook was professionally translated into the Spanish language and iteratively refined via review from Spanish speakers from multiple countries of origin to ensure comprehension.

During intervention sessions, facilitators combined education about cGVHD and survivorship with training in self-management skills. Dedicated time was provided for participants to share experiences and receive social support. Between sessions, participants were encouraged to set practice goals and to discuss any questions about their medical history or laboratory results with their HCT clinicians, reinforcing empowerment and self-management. Session topics included an introductory session; managing symptoms and quality of life; coping with uncertainty; practicing self-care; navigating intimacy and relationships; exploring meaning in experiences; and looking ahead to the future. Co-leaders facilitated participant discussion of individual, family, social, and cultural experiences related to the program’s medical information and coping topics. Details on session content have been previously reported [18].

### Measures

#### Feasibility.

We tracked enrollment, attendance, and retention rates, recruitment methods, and reasons for non-enrollment or attrition. We specified a priori benchmarks for feasibility based on prior supportive care work with transplant recipients (e.g., [21]). Benchmarks included enrollment of ≥50% of patients approached, ≥80% attendance in at least half (4/8) of the Horizons program sessions, and retention, with ≥80% completing follow-up assessments.

Interventionists participated in virtual training and attended weekly Zoom-based team consultations with the lead licensed psychologist, who provided oversight (L.T.). We also audio-recorded intervention sessions and reviewed 100% of sessions. After each session, study staff present completed brief surveys to report session duration, attendance, and observational notes. Four study team members (A.P., C.W., J.C., J.E.) independently reviewed the Spanish- and English-language audio recordings and rated adherence to the intervention content using a prespecified list of intervention topics, one per session.

#### Acceptability.

At 10 weeks post-intervention, study staff additionally administered the Client Satisfaction Questionnaire (CSQ-8; range of 8–32 with higher scores indicating greater satisfaction) to measure acceptability regarding aspects of the intervention (e.g., perceived quality, perceived helpfulness) and the Group Cohesiveness Scale (GCS; range of 35 with higher scores indicating greater cohesion) to measure perceived cohesion within participants’ intervention group [22–23].

We developed a semi-structured exit interview guide for participants that included questions and probes addressing motivations to participate; perceptions of program content, structure, linguistic adaptation, cultural relevance, program benefits, and group dynamics; and recommendations to improve the program. The interview guide was developed and cognitively tested by two authors (L.T. [psychologist, female] and J.C. [graduate student, female]) with experience in qualitative research. The exit interview guide was professionally translated into Spanish language and refined via review from native Spanish speakers to ensure understandability. All participants were invited to complete an exit interview to ensure representation of those who completed all intervention sessions and those who did not. At the 10-week time point, trained study staff members (English: J.C. and M.P.; Spanish: J.E. and J.M.) conducted each 1:1 exit interviews, in English or Spanish language, via teleconference. Throughout each audio-recorded interview, study staff conducted respondent validation by restating a summary of each response and providing an opportunity for clarification to enhance rigor in subsequent data interpretation. Exit interviews were transcribed and, if conducted in Spanish, translated by study staff for analysis.

#### Self-Report Assessments.

All participants completed baseline and follow-up self-report assessments at 10 weeks post-intervention initiation, in English or Spanish language (i.e., via professional translation or the publisher’s translation), administered by bilingual study staff in person, by phone, or via secure email. Participants completed a demographic questionnaire (e.g., age, gender, race, ethnicity, education, relationship, and living status) at baseline. Clinical information (e.g., diagnosis, date of transplant) was collected from the electronic health records. We used six questionnaires to guide our future trial: anticipated program outcomes (i.e., quality of life, symptom burden, and psychological distress) and self-management targets (self-efficacy, coping skills, and social isolation). The Functional Assessment of Cancer Therapy–Bone Marrow Transplant Scale (FACT-BMT) assessed QoL in the past week [24–25]. Responses are summed to yield a total score; higher scores indicate better QoL. The Lee Chronic GVHD Symptom Scale measured patient-reported cGVHD symptom burden [26]. Scores range from 0 to 100; higher scores indicate worse symptoms. The Hospital Anxiety and Depression Scale (HADS) assessed mood symptoms [27–28]. The 14-item HADS consists of two subscales that capture anxiety (HADS-A) and depression (HADS-D) symptoms in the past month. Subscale scores on the HADS range from 0 to 21 (maximum distress level), with scores greater than 8 indicating clinically significant symptoms on the respective subscale. The Cancer Self-Efficacy Scale-Transplant (CASE-T) measured confidence in managing the impact of cGVHD [29–30]. The CASE-T consists of 17 items; higher scores indicate greater self-efficacy. The Measurement of Current Stress (MOCS) assessed perceived coping skills [31–32]. The MOCS consists of 13 items; higher scores indicate greater coping skills. The 4-item PROMIS-Social Isolation scale (PROMIS-SI) assessed social isolation; higher scores indicate greater social isolation [33–34]. Demographic survey items, Group Cohesiveness Scale, Cancer Self-Efficacy Scale-Transplant, and the Lee Chronic GVHD Symptom Scale were professionally translated for the present study, as Spanish language versions were unavailable at the time of study conduct.

#### Data Analysis

We performed all quantitative analyses using R and RStudio Version 4.4.2. Descriptive statistics were used to summarize sample characteristics and feasibility outcomes. We used Pearson’s correlations and independent samples t-tests or one-way analysis of variances to explore relationships of continuous and categorical sample characteristics with attendance and retention feasibility outcomes. We analyzed the open-ended exit interview data using a content analysis approach, to help further characterize feasibility and acceptability. We first derived codes deductively from the interview guide topics of feasibility, acceptability, critiques, and perceived benefits. We then reviewed a sample of interview transcripts to gain familiarity with the content and generated additional descriptive codes inductively based on the raw exit interview data. Subsequently, the transcripts were examined line by line, and meaningful segments were assigned codes that captured the essence of participants’ statements. This process was iterative, with codes refined, merged, or expanded as five study staff piloted the formulated codebook across a subset of transcripts (A.P., E.H., J.C., J.E., H.C.). Once the codebook was finalized, two study staff (J.C., S.B.) independently coded, using NVivo 15, all transcripts to enhance reliability, and discrepancies were resolved through discussion. We then summarized the coded feedback on Horizons program’s feasibility, acceptability, critiques, perceived benefits, and other emergent topics [35–36], organized the summaries into key categories, and assigned descriptive names to each category. Illustrative quotes were selected to reflect each category.

## RESULTS

### Feasibility

#### Recruitment, Enrollment, and Retention.

**Figure 1:**
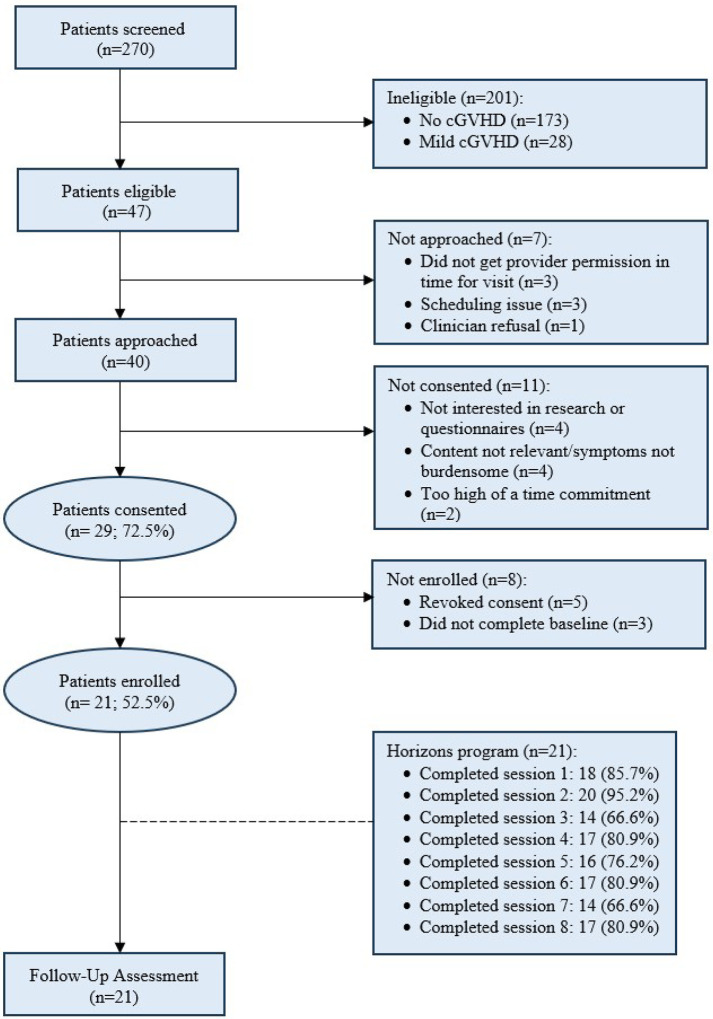
Study CONSORT Diagram

We approached 40 patients for study participation between December 2023 and August 2024, and 72.5% (29/40) provided consent (Figure 1). Of 29 consented participants, 8 were not enrolled due to not completing the baseline survey (n=3) or revoking consent (n=5), and 21 (52.5%, 95% CI [37.5%, 67.1%]) completed enrollment. Ten participants were assigned to the English-language groups and 11 to the Spanish-language groups, based on participant preference. Of 21 enrolled participants, 95.2% (n=20; 95% CI [77.3%, 99.2%]) completed follow-up assessments at 10 weeks.

Sociodemographic characteristics of the sample are reported in Table 1. Of the 21 participants, 71% (n=15) identified as male and nearly three-quarters (n=15) as White. Fifteen (71%) also identified as Hispanic/Latino, indicating that even the English-language groups were majority Hispanic/Latino. Most were unable to work due to illness (n=12) which is representative adults with cGVHD [37]. The median age was 60 years (range 22–74). Clinically, 10 participants had moderate cGVHD, and 11 had severe cGVHD. Median time since allogeneic transplant was 54.1 months (*range* 9.6–222), and median time living with CGVHD was 34.3 months (range 1–216.3). At baseline, 19.0% reported clinically significant anxiety symptoms (n=4) and 23.8% depression symptoms (n=5). Overall, only 5.4% of data was missing.

#### Session Attendance.

Nineteen participants (90.5%; 95% CI [37.5%, 67.1%) attended at least four of eight sessions. Sessions were most commonly missed due to scheduling conflicts (n=7), medical appointments and illness (n=6), and personal travel (n=6).

#### Fidelity.

Treatment fidelity, defined as the coverage of at least 80% of material per session, was 96.7% on average (range, 85.7%–100%).

### Acceptability

#### Motivations to Participate:

In exit interviews, participants commonly acknowledged initial apprehensions about study participation, such as concerns about session length or risk to personal privacy, or a sense that they would not be a good program fit due to their cGVHD symptom profile or other factors. Yet participants, particularly Spanish-language participants, felt motivated by the opportunity to help others and to raise awareness of cGVHD, and subsequently expressed pride in contributing to a resource for people living with cGVHD.

#### Group Cohesion and Kinship:

Group cohesion scores (M=31.80 [SD = 3.35]) suggested relatively high levels of perceived cohesion among group members. Shared trust and comfort developed over time, with one participant emphasizing that they “*were all [in Horizons] together for the same thing*” (ID 122). Participants commonly noted that their participation in the Horizons program was the first time they connected with others who understood life after transplant. Several participants highlighted that even medical professionals could not fully grasp what it was like to live with cGVHD, making peer connections particularly validating and comforting. Some participants felt responsible for showing up to sessions to comfort each other. One participant expressed, *“There are people in the study [that] have worse [symptoms] than others, and there [are] some people that have less [symptoms] than others. I think at the end of the day, everybody was still trying to comfort each other, no matter how difficult [their] experience has been so far with the disease” (ID 116)*. In both English and Spanish language groups, the **sense of community** (e.g., “*being in the same boat*”) was commonly identified as **the program’s most important and helpful aspect**, offering benefits such as emotional relief, companionship, and a space to feel **understood**. Among English- and Spanish-speaking participants, many reported that cGVHD was a unifying experience that transcended national and cultural boundaries. However, Spanish-speaking participants commonly expressed a heightened sense of connection and comfort within their groups. Several participants noted that their Spanish-language group enabled them to openly acknowledge culture-specific experiences through personal anecdotes shared during group sessions.

#### Learning through Others:

Differences in various clinical and demographic factors (e.g., cGVHD symptom severity, cGVHD stage, time since diagnosis, age, gender) generated initial discomfort for some participants (e.g., sense of ‘intruding’ on group members with more severe symptoms) while also presenting critical learning opportunities over the course of the program (e.g., gaining insights from long-term survivors or gaining opportunities to guide and support newer survivors). Participants found value in hearing about others’ coping strategies and future planning, which prompted them to reflect on how they might apply similar approaches in their own lives. One participant acknowledged that it was helpful to have a space for “*communication of [ideas], rather than just thinking”* in isolation (ID 115).

#### Grasping New Knowledge and Skills:

Client Satisfaction Questionnaire scores demonstrated high satisfaction with the Horizons program (*M* = 29.75, *SD* = 2.59). English- and Spanish-language participants consistently reported gaining new knowledge about cGVHD and its management. Many described increasing awareness of cGVHD as a chronic condition. In the context of the evolving treatment landscape for cGVHD (“*It is a very difficult disease that we do not completely understand*” [ID 115]), the opportunity to learn about various symptom-management strategies from clinician co-facilitators and other group members was perceived as a valuable component of the program. Several participants also highlighted various coping strategies (e.g., mindfulness exercises) that were helpful to learn and apply to stressful situations.

### Recommendations

Participants offered several recommendations to enhance the Horizons program with respect to program structure. Recommendations regarding structure commonly reflected a desire for more engagement opportunities. Some participants wanted more flexibility and time to allow group members to bring forward areas of personal relevance and/or deepen their connections with each other. Participants also commonly wished for opportunities to sustain connections with the other group members beyond the program. A few Spanish-speaking participants, in particular, recommended including family members, noting that their involvement in group sessions could enhance support. Finally, several participants expressed interest in having an abbreviated version of the program workbook as a resource outside of sessions. Feedback on program timing, relative to the time course of participants’ cGVHD, was mixed. Some participants felt that participating earlier after their cGVHD diagnosis might better support their adaptation and coping. Yet, including individuals at different ranges of time since cGVHD diagnosis was suggested to be beneficial, as those with long-term experience can provide guidance and perspective.

## DISCUSSION

This mixed-methods pilot study examined the feasibility and acceptability of the Horizons program among English and Spanish-speaking patients. Supported by both quantitative metrics and qualitative feedback, our findings demonstrate that the Horizons program, delivered remotely in both languages, is a feasible and valued intervention, extending the results of prior pilot testing [18, 19]. Prior qualitative findings indicated that the Horizons program was perceived as an important opportunity for patients to enhance social support, self-efficacy in managing cGVHD, and motivation to achieve self-care goals in an English-speaking sample [19]. In the current study, hypothesized feasibility benchmarks for enrollment, retention, session attendance, and fidelity were surpassed, supporting program delivery in English and Spanish languages and aligning with behavioral medicine principles of interventions being patient-centered, accessible, and supportive.

A key driver and benefit of participation was the opportunity to connect with peers facing similar challenges, alleviating the social isolation often experienced by cGVHD patients due to prolonged immunosuppression and physical limitations documented in prior research (e.g. [36, 38]). Individuals living with cGVHD may seldom encounter someone else living with their condition. Participants’ perceptions are consistent with social cognitive processing theory and prior work indicating that open communication and social support promote adaptation and psychological well-being in cancer survivorship [39–40]. Participants described our multidisciplinary co-leadership model as effective in normalizing diverse survivorship experiences and fostering group cohesion among participants spanning a wide range of symptom severity, disease duration, and sociodemographic backgrounds. A typical non-specific benefit identified in the participant feedback was strong group dynamics, which generally includes cohesiveness and bonding [41]. This pattern echoes prior research illustrating that group dynamics are crucial to behavioral interventions focused on psychosocial adjustment in individuals with chronic illnesses [42]

While not a primary motivator for many participants, skill acquisition emerged as a critical benefit, highlighting the need for structured psychoeducation, self-management training, and peer exchange to enhance self-efficacy and QOL in cGVHD. Participants recommended flexible enrollment across the disease trajectory, valuing mutual mentoring between newly diagnosed and long-term survivors, which may optimize both learning and peer support. In doing so, this program model addresses documented gaps in survivorship care among HSCT populations [43].

The overall experiences shared by participants in the Horizons program revealed that cGVHD was a largely unifying experience across both English- and Spanish-speaking groups. Spanish-speaking participants emphasized the importance of cultural connection and comfort, noting that using Spanish created a culturally safe space that enhanced openness and identification. This contrast may reflect the importance of linguistically tailored interventions in fostering a sense of belonging and psychological well-being. These findings align with existing research demonstrating that shared illness experiences can transcend cultural boundaries; however, culturally responsive group settings enhance engagement and support [44–45]. Recognizing both the universal impact of cGVHD and the role of culture informs future program development to optimize support for diverse survivor populations. Spanish-speaking participants’ emphasis on the lack of awareness of cGVHD and support for this condition may also reflect a need for greater inclusion of individuals whose primary language is not English.

Results should be interpreted with attention to study limitations. Recruitment from a specialized transplant survivorship clinic may limit generalizability to transplant recipients without such dedicated access to care. The data were also obtained from participants in a pilot single-arm trial and may not reflect the perspectives of patients who declined to participate. Additionally, the program’s age-related experiences were not specifically probed. Older adults living with cGVHD often face greater comorbidity and functional burden, though they demonstrate preserved QOL and similar overall survival and non-relapse mortality when compared to younger patients [46–48]. Younger adults navigate disruptions to work, identity, and long-term survivorship [49–50]. These differences influence participants’ psychosocial needs and their engagement with and benefit from a coping intervention. Future work should also emphasize multisite designs, including those involving underrepresented racial and ethnic populations, and assess hybrid delivery options that combine telehealth with optional in-person engagement to accommodate varied preferences, consistent with the telehealth behavioral intervention literature [51].

## CONCLUSION

In conclusion, the Horizons program represents a feasible, acceptable, and valued multidisciplinary group coping intervention for English and Spanish speakers addressing social isolation, psychosocial distress, and self-management challenges among adults with moderate or severe cGVHD. By fostering peer connections, enhancing coping skills, and promoting patient empowerment, this intervention aligns with the objectives of behavioral medicine to improve chronic illness survivorship outcomes. The present data provide foundational support for scaling this intervention in a larger randomized controlled trial to evaluate clinical effectiveness, linguistic adaptation, and implementation across diverse transplant survivor populations.

## Figures and Tables

**Table 1.** Participant Demographics and Clinical Characteristics (N=21).

Variables	Current Study Sample
	(N=21) *n (%)*
**Age** (Median, range)	65.5 (22 – 74)
**Gender**	
Man	15 (71.4)
Woman	6 (28.6)
**Preferred Language**	
English	11
Spanish	10
**Ethnicity**	
Hispanic/Latino	15 (71.4)
Non-Hispanic	6 (28.6)
**Race**	
White	15 (71.4)
Other (Not Listed)	6 (28.6)
**Relationship Status**	
Married or in a Non-cohabitating Relationship	12 (57.1)
Single, never married	5 (23.8)
Divorced/Separated	2 (9.5)
Widowed/Loss of Long-term Partner	2 (9.5)
**Education**	
High School Diploma (GED) or less	6 (28.6)
Some college/college degree	10 (47.6)
Post-graduate, professional, or doctorate	5 (23.8)
**Employment**	
Employed (Full or Part Time)	8 (38.1)
Unemployed or Retired	2 (9.5)
Unable to Work due to Illness/Disability	12 (57.1)
**Household Income**	
<$25,000 – $50,000	14 (71.4)
$50,000 – $100,000	3 (14.3)
>$100,000	3 (14.3)
Missing	1 (4.7)
**Diagnosis**	
Acute Leukemia	10 (47.6)
Lymphoma	2 (9.5)
Myelodysplastic Syndromes	5 (23.8)
Other	4 (19.0)
**Months Since HCT** (Median, range)	54.1 (9.6 – 222.0)
**Months Living with cGVHD** (Median, range)	34.3 (1.0 – 216.3)
**Chronic GVHD Severity**	
Moderate	10 (47.6)
Severe	11 (52.4)
**Anxiety Symptoms**	4 (19.0)
**Depression Symptoms**	5 (23.8)

## Data Availability

The data are available upon reasonable request, subject to the permission of the corresponding author’s institutional IRB.
